# Editorial: The interaction between the oral microbiota and systemic diseases

**DOI:** 10.3389/fdmed.2024.1390188

**Published:** 2024-04-09

**Authors:** Camila Lopes Crescente, Marinês Nobre-dos-Santos, Fabíola Galbiatti Carvalho, Ramiro Mendonça Murata, Lívia Araujo Alves, Thaís Manzano Parisotto

**Affiliations:** ^1^ Laboratory of Clinical and Molecular Microbiology, University São Francisco, Bragança Paulista, Brazil; ^2^ Department of Pediatric Dentistry, University of Campinas, Piracicaba, Brazil; ^3^ Department of Pediatric Dentistry, Federal University of Uberlância, Uberlândia, Brazil; ^4^ Department of Foundational Science, ECU School of Dental Medicine, Greenville, NC, United States; ^5^ Odontology Post Graduation Program, University of Cruzeiro do Sul, São Paulo, Brazil

**Keywords:** bacteria, oral health, systemic disease, microorganism, humans

**Editorial on the Research Topic** The interaction between the oral microbiota and systemic diseases

The oral cavity harbors one of the human body's most diverse and complex microbial communities and is usually in homeostasis with the host. However, dysbiotic conditions can lead to severe oral and systemic infections. Interestingly, many systemic conditions manifest in the mouth, exacerbating oral diseases and highlighting the oral cavity's role as an indicator of systemic disturbances.

The present research topic comprises five published papers: two studies involving human subjects, one animal, and two mini-reviews from North and South American researchers at schools of Medicine, Dentistry, and Biomedical Sciences. The comprehensive mini-review entitled “Oral dysbiosis and systemic diseases” (Georges et al.) explores the connection between metabolic diseases (diabetes mellitus and cardiovascular problems) and immunologic conditions (Lupus and Sjogren's syndrome) with oral microbial dysbiosis. The work of Georges et al. reveals a strong correlation between dysbiosis in the oral environment and the pathogenesis of systemic diseases. For instance, the dysbiosis of the mouth environment may exacerbate the pathogenesis of systemic diseases. In this sense, periodontal disease could be worsened as a consequence of unbalanced type II diabetes. Similarly, other inflammatory/autoimmune systemic problems might favor xerostomia, mouth mucositis, mucosal ulcers, and enhanced susceptibility to oral infections (Georges et al.).

Another significant contribution to this research topic emphasizes that changes in the oral microbiome resulting from shifts in an individual's lifestyle can extend beyond local impacts on oral tissues, potentially interacting with intestinal microbes and influencing systemic environments (Reis et al.). An example of a substantial lifestyle change was the COVID-19 pandemic, with social isolation enforced as a protection measure. This period posed a particular challenge for children. As suggested in the mini-review by Reis et al., “*Possible Relationship Between the Oral and Gut Microbiome, Caries Development, and Obesity in Children During the COVID-19 Pandemic*”, environmental and social factors can significantly impact the health-disease process mainly because of changes in the dietary habits and the immune system, culminating in diseases such as caries and obesity.

Furthermore, within the context of COVID-19, investigations have explored changes in oral/nasopharyngeal bacteria during and after SARS-CoV-2 infection, comparing them with those in healthy individuals (Callahan et al.; 1, 2). The salivary microbiome, known to be relatively stable in adults, could change when exposed to SARS-CoV-2. In addition, the virus impacted abilities/characteristics of the oral environment, including loss of taste, dry mouth, dysgeusia, and opportunistic infections that can alter the microbiota. In this context, an original retrospective pilot study entitled “Oral microbial taxa associated with risk for SARS-CoV-2 infection”, conducted at the University of Illinois—Chicago at the College of Dentistry, noted that salivary bacteria taxa might be associated with an increased risk of SARS-CoV-2 infection in the unvaccinated population (Callahan et al.).

The paper titled “Diet-induced non-alcoholic fatty liver disease and associated gut dysbiosis are exacerbated by oral infection” (Simas et al.) highlights the association between oral infection by *Porphyromonas gingivalis*, a prominent agent in periodontal disease, and the progression of non-alcoholic fatty liver disease (NAFLD). Exploring this relationship, this relevant investigation used a rodent model to demonstrate that infection by *Porphyromonas gingivalis* produces a persistent alteration in the composition of intestinal microbiota, reinforcing the intimate connection between the oral environment, the resident microbiota, and the general systemic condition.

The final study, “*Detection and quantification of pathogens in the saliva of adolescents with cerebral palsy: a cross-sectional study involving adolescents*” (Yoshida et al.), emphasizes the variability in DNA detection of salivary periodontopathogens in youths with cerebral palsy, particularly considering the presence of *Porphyromonas gingivalis* in individuals with gingivitis. It is common for patients with cerebral palsy to experience gingival inflammation. Gingivitis precedes periodontitis, a disease of challenging treatment, as it relies intimately on the patient's compliance and requires close periodic monitoring. In the case of cerebral palsy, the caregiver's compliance in maintaining the patient's good oral hygiene, together with their already extensive workload, is not easy.

All the exciting manuscripts mentioned above were published in Frontiers in Dental Medicine (2022–2023) in “*The Interaction between the Oral Microbiota and Systemic Diseases*” special topic. The significant scientific findings enrich our understanding of the complex relationships between oral microbial communities, their surrounding environment, and systemic diseases. We believe this topic provides critical evidence for developing innovative research strategies that not only involve the crosstalk between the oral microbiome and systemic diseases but also encompass various dimensions, including the dysbiosis/host immune response axis, and social determinants of health.
